# Qualitative analysis of the Best Possible Self intervention: Underlying mechanisms that influence its efficacy

**DOI:** 10.1371/journal.pone.0216896

**Published:** 2019-05-17

**Authors:** Alba Carrillo, Marian Martínez-Sanchis, Ernestina Etchemendy, Rosa M. Baños

**Affiliations:** 1 Department of Personality, Assessment and Psychological Treatments, University of Valencia, Valencia, Spain; 2 Department of Psychology and Sociology, University of Zaragoza, Teruel, Spain; 3 CIBER Fisiopatología Obesidad y Nutrición (CIBEROBN), Instituto Carlos III, Madrid, Spain; University of Auckland, NEW ZEALAND

## Abstract

**Background:**

The Best Possible Self is a Positive Psychology Intervention which asks participants to write down about themselves in their best possible future. Previous studies have shown its efficacy to enhance wellbeing, but the mechanisms that underlie its efficacy are still unknown.

**Objective:**

The aim of this study was to analyze the content of the essays of the BPS intervention and to examine how this content was related to the efficacy of the intervention to increase positive affect.

**Method:**

Participants (N = 78) were randomized to either the Best Possible Self condition, or one of two variants of the intervention: one’s best self in the present, and one’s best self in the past. Qualitative analyses of the texts were carried out to explore the main themes and features of the essays. Then, a mixed-methods approach with quantitative and qualitative data was followed, in order to analyze the relationship between the content of the texts and the change in positive affect produced by the interventions.

**Results:**

Significant differences between conditions were found in the content of the compositions. Regression analyses showed that different variables predicted the change in positive affect depending on the condition. Mediation analyses also found differences among conditions.

**Conclusions:**

These findings suggest that these interventions respond to different underlying mechanisms which influence their efficacy. This study contributed to a better understanding of how Positive Psychology Interventions work, and how to increment their efficacy.

## Introduction

Historically, individuals have made profuse efforts to achieve the road of happiness and wellbeing. Lately, these efforts have crystallized in the Positive Psychology research movement, whose aim is to provide an evidence-based framework for the study of what makes people happy and how to bolster their wellbeing levels [1]. Although there is a lack of a unified definition of wellbeing, one of the main historical approaches proposes wellbeing as the balance between positive and negative emotions and a high sense of satisfaction with life, also known as subjective wellbeing (SWB) [2]. Positive Psychology Interventions (PPIs) emerged precisely as a response to the societal need of increasing people’s overall wellbeing levels, including SWB. This applied portion of Positive Psychology consists of activities aimed at increasing positive emotions, cognitions or behaviors [3,4]. Research on the efficacy of these interventions has burgeoned since its beginning, and nowadays there are multiple published studies about new and heterogeneous exercises that can help people flourish (e.g. gratitude letters, acts of kindness, using signature strengths). Indeed, several meta-analyses have shown that PPIs are effective approaches to increase wellbeing with small to moderate effect sizes [4,5].

Lately, as a consequence of the progression in the knowledge of these interventions, research interest on the mechanisms that explain the efficacy of PPIs is growing. To date, some authors have attempted to explain why and under which circumstances PPIs work, developing some theoretical models that can be applied to all PPIs in general (e.g. [6,7]). However, this field is considerably recent, and these models still need to be validated [8]. In addition, they are applied to the complete range of PPIs despite their heterogeneity, hence there is a lack of knowledge about the circumstances that make each intervention individually effective [5,6].

One of the most widely used PPIs is the Best Possible Self (BPS) intervention, in which participants are asked to write down about their best possible self in a future where they have achieved everything desired, after working hard towards it. This intervention was developed initially by King [9], and it was based on the trauma writing paradigm, which had found that writing sessions about upsetting and negative topics (as a traumatic event) produced both physical and mental health improvements [10,11]. As a response to the emerging interest in the positive side of life [1], the focus of attention in research changed from the trauma writing paradigm to the positive writing paradigm (i.e., writing about positive topics) and its effects on wellbeing, being the BPS intervention one of its main examples. Based on the writing paradigm of Pennebaker, King [9] developed this intervention and compared it with a writing disclosive exercise about a traumatic event. Results showed that BPS intervention produced the same benefits as trauma-focused writing on health. This intervention, in addition, produced significant increases in positive mood and wellbeing, and participants in this condition rated the exercise as less upsetting than the trauma condition participants. These results are consistent with the last meta-analysis about disclosive writing, which found no significant differences between interventions focused on disclosing negative events and the ones focused on disclosing positive events on psychological and health benefits [10]. As the author of this work stated, given that trauma writing paradigms usually produce temporary increases in negative affect, choosing the disclosure of positive events may be preferable, as it avoids this short-term negative side effects and it has shown the same positive results.

Since the first approach by King, many studies have been carried out in order to test the efficacy of this PPI. A narrative review of this intervention concluded that it seems a viable approach to produce positive outcomes on wellbeing, although little is known about how this positive activity works [12]. In addition, a recent meta-analysis about 28 studies showed that BPS is an efficacious intervention to improve wellbeing and found moderate effect sizes of BPS over control groups on positive affect (d = .339 and d = .657) [13]. However, analyses of moderators (i.e., length, dosage, delivery method, etcetera) did not show significant results in this review. Therefore, the characteristics of the BPS intervention that might influence its efficacy are still unknown.

A methodological approach that has potential to unveil the possible mechanisms that underlie the efficacy of a writing intervention is a qualitative analysis of its content. This approach has the ability to uncover novel and deeper understandings of phenomena of interest in Positive Psychology [14]. In addition, when combined with quantitative data in a mixed-methods approach, qualitative data can help to identify significant predictors of wellbeing, producing a more comprehensive outlook of relevant constructs and addressing questions as *why* and *how* [15].

Recently, the benefits of positive writing have derived to an increasing interest on the qualitative variables of the writing tasks, although research is still scarce. In the case of the BPS intervention, only a handful of studies explored the content that participants wrote about. King [9] found that the BPS essays included a variety of topics, such as job success, self-improvement, marriage and family, travel, or home ownership, although no further analyses were carried out on the frequency of these topics. Hill and colleagues [16] analyzed the texts of the BPS compositions in order to classify the goals included in the essays and found fourteen categories. The most frequent goals were *approach* (those with references to approaching something positive), *intrapersonal* (goals that mentioned only the self), and *achievement* (those goals related to accomplishing a goal or achieving success). Correlation analyses were carried out to explore the association between written goals and measures of life satisfaction and religiosity. Results showed that life satisfaction was negatively correlated with *spirituality* goals (related to a higher power and/or to unity and justice). In addition, Loveday et al. [17] carried out a thematic analysis of the BPS texts specifically focused on spare time using an explicit conceptual framework on leisure [18]. Results showed that within the leisure area, *affiliation* (leisure spent with other people), *autonomy* (leisure spent on oneself) and *detachment-recovery* (leisure mentioned in relation to work) were the most frequent themes (33, 23, and 21 percentage of leisure sentences, respectively). However, this study only addressed the content of the essays within the previously mentioned framework–focused on spare time, and only analyzed the sentences coded as *leisure*, which represented 41% of the content, whereas the remaining 59% of the sentences categorized as non-leisure were not explored. As it can be seen, these first approaches have explored the qualitative characteristics of the texts of the BPS essays, but they were carried out within specific frames that might have constrained their results. Hence, there is still a scarce knowledge about which content, in general, participants include in their essays when they write about their BPS, and a broader approach could contribute to a better understanding about this subject. In addition, none of these studies have combined the content analyses with quantitative data about the efficacy of the intervention, thus the role that the content of the texts may play on its efficacy is still unknown.

This study is part of a larger project on the mechanisms that underlie the efficacy of the BPS intervention [13], in which the role of temporality was explored. A randomized controlled trial with three experimental conditions (the original BPS and two temporal variations: past best self or BPAS and present best self or BPRES) showed that temporal focus did not affect the ability of the intervention to increase positive affect as no statistically significant differences emerged among conditions. Therefore, it is necessary to continue investigating on the underlying mechanisms that influence the efficacy of the BPS.

The main objective of the present work was to analyze the role of the content of the essays of the BPS intervention on its efficacy. For this purpose, qualitative analyses of the BPS and the BPRES and BPAS variants were carried out. More specifically, this work had two aims. On the one hand, to analyze the content of the texts in order to identify the main themes and features of the compositions of the three PPIs (BPAS, BPRES, BPS) and to explore the possible differences between conditions. On the other hand, to examine the influence that the identified themes and features of the texts had on the efficacy of the interventions to increase positive affect. As far as we know, this is the first study that systematically analyzes the content of the texts of the BPS with a bottom-up approach (not forced by a predetermined model), and the first one that includes the temporal variants of this intervention. In addition, this is the first work that combines qualitative data of the interventions with quantitative data about their efficacy. It was expected that the content of the texts would influence on the efficacy of the three PPIs to increase positive affect. However, due to the exploratory nature of the analyses, no specific hypotheses were generated regarding the content of the texts on each intervention and how it may affect the efficacy of the intervention.

## Method

### Participants

The initial sample consisted of 84 participants who were part of a larger study [13]. Their age ranged from 18 to 40 years old (M = 20.23, SD = 4.10), and 77.2% of them were women. They were randomized to one of three conditions: BPAS (N = 30), BPRES (N = 27), BPS (N = 27). Two participants did not answer post-intervention assessment, and text analyses showed that four participants did not follow the instructions of the assigned conditions. Consequently, six participants were excluded from the study. The final sample consisted of 78 participants (BPAS = 27, BPRES = 25, BPS = 26).

### Interventions and procedure

This study included three PPIs, based on the original BPS exercise. The BPS intervention asks participants to visualize themselves in the future after everything has gone as well as possible [19,20]. Based on this intervention, two variants of the exercise were designed with the same format and instructions, except for the time frame in which they were focused on. Concretely, the Best Past Self condition (BPAS) required to recall a time in the past when participants considered they had displayed the best version of themselves, whereas the Best Present Self condition (BPRES) asked participants to think about the best version they offered to the world at the present time.

The procedure was based in previous studies: participants were encouraged to write about their best self and then to mentally visualize this content [19,21,22]. The complete intervention lasted 7 days, in which participants came to the laboratory for the first session and then practiced the assigned exercise at home for one week. During the first session, participants signed the informed consent, answered the pre-intervention assessment and listened to audiotaped instructions of the assigned task. Regardless of the condition, they had to spend 15 minutes writing their essay in a computer in the laboratory, and then 5 minutes visualizing its content (their best self) [19,23]. Instructions in all conditions encouraged participants to include as many sensorial details as possible, as the procedure included an explicit visualization component in which they spent 5 minutes visualizing about their best self after writing about it [19,23]. During the remaining 6 days, participants were encouraged to mentally visualize the content of their essay once a day. After 7 days, participants received a link with the post-intervention assessment.

This work was registered in the United States National Institute of Health Registration System (http://www.clinicaltrials.gov) with Clinical Trials Registration Number NCT03024853 and approved by the ethical committee of the University of Valencia (H1415802387094).

### Scales

A mixed-methods approach using quantitative and qualitative methodologies was followed in order to explore the relationship between the content and features of the texts and the change in positive affect.

The quantitative outcome measure included was positive affect, as it has been widely used in previous studies (e.g. [19,20,24]). The scale used to measure positive affect (PA) was the PA subscale of the Positive and Negative Affect Scale, PANAS [25], which includes 10 positive emotions (e.g., inspired) to measure positive mood. Respondents rate how they usually feel on a 5-point Likert-type scale. In this study, a Spanish version was used [26].

Cronbach’s alpha for the original scale ranged from .86 to .90, and in this sample alpha value was .90. Participants answered the scale the first day before practicing the assigned exercise (pre-intervention assessment), and 7 days after the intervention started (post-intervention assessment).

### Coding of the essays

Essays were analyzed to explore two main areas. On the one hand, the content of the essays (i.e., what did participants write about when they reflected on their best past, present or future self). On the other hand, the features of the compositions or, in other words, how they expressed these ideas (e.g., the number of words or its emotional valence).

The followed approach was based on the consensual qualitative research-modified (CQR-M), a qualitative research method designed to be applied in large samples (i.e., more than 15 participants) and relatively brief qualitative data, which can be used to describe little-studied phenomena and establish a basis for further research. This method is defined as a bottom-up approach, through which categories are derived from the data instead of forcing a predetermined structure on it [27]. With this method, as the authors state, a further comprehension of the topic under research can be obtained by combining the newly described phenomena with quantitative data.

In order to reach consensus, following the CQR-M guidelines, all team members discussed disagreements at each step of the process. The coding team was composed by the first and second authors (AC and MMS), who were experts on the interventions used in the study, knew the instructions and procedure and had previously conducted studies with the included activities. The next procedure was followed: first, two independent coders (AC and MMS) read all the essays independently and generated a list of themes and areas identified in the texts. Secondly, these themes were discussed by the researchers, and then the revised themes were applied in the analyses of 30 randomized essays, in order to explore whether these were adequate and captured all the relevant ideas. After a revision of the themes, all essays were analyzed independently by the two coders in order to categorize all the contained bins of information with the designated themes and the subsequent areas. Interrater reliability and frequency of themes were calculated (see Method and Results sections).

These themes were not mutually exclusive. In addition, since this analysis relied on bins of information, they did not necessarily coincide with a complete sentence: it was possible that a single sentence contained two ideas (for example, “the social area is very important in my life: I like to communicate with people and I tend to be quite open and affectionate”, would be coded as *friendship* and *positive features*), and it was also possible that the same idea expressed in two or more sentences would be coded as one unit (for example, the two sentences “I want to expose myself to what life brings to me. I want to feel inexperienced to able to improve” would be categorized as *positive features*).

#### Themes of the texts

The final categories included could be grouped into four areas: personal, academic/professional, social, and leisure area. Regarding personal area, *positive features* collected all phrases that expressed a personal improvement on one’s trait or psychological ability, or an already present positive feature that remained constant (e.g., “In the future I would like to have the same psychological abilities that I currently have”); *skills* referred to the presence or the willingness to learn an ability or knowledge (e.g., “I would like to learn how to play the piano or the harp”); and *health* was coded when participants talked about their attempts to influence their physical health (e.g., “My best self figured out my intestinal problem and now she’s thin and strong”). Concerning the academic or professional area, themes were divided by the inner motives expressed in the texts, being *intrinsic* the content related with the academic or professional area associated with intrinsic motives (e.g., “Now I have a job in which I feel very happy, and I have realized that I love my job”), and *extrinsic* when extrinsic motives were expressed (e.g., “I visualize myself wearing a suit and having quite a lot of money”). With respect to the social area, *friendship* was coded on phrases containing social relationships with friends or colleagues (e.g., “I felt very close to my childhood friends because we were all going through the same phase”), *family* on mentions to relationships with members of the family (e.g., “When I get home, I tell my family about my day and I hear about theirs”), *partner* in the case of romantic relationships (e.g., “I had a partner with whom I enjoyed our shared moments”), and *help* emerged when participants made an explicit reflection on their willingness to or their actions aimed at helping other people in different contexts (e.g., “I decided I would watch over the happiness of others, trying to improve their lives”). Lastly, the leisure area only included the *leisure* theme, which contained phrases related to how their best selves spent their free time or practiced different hobbies (e.g., “I had time to watch TV series and movies”).

#### Features of the texts

In addition, the collected features of the compositions were: length of the essay (total number of words), quantity of sensorial details (e.g., “I was drinking tea, it tasted stronger than usual. I added sugar and started to blow, it was so hot … I could see the steam coming out of the cup”), emotional valence of the essay, and incongruousness. Emotional valence was calculated as the subtraction of the total number of positive emotional states (e.g., “It was some years ago, but the feeling still lingers: pride”, “I feel vigorous, energetic, tolerant and strong”) minus the number of negative emotional states (e.g., “In my future I keep seeing a lot of stress and anxiety”, “I feel pretty demotivated in my academic life”) in each text. Regarding incongruousness, it was coded on phrases in which participants talked about a positive feature explicitly expressed as no longer present (e.g., “I have the feeling that I enjoyed the little things more than I do now”), or the willingness to reduce or eliminate the presence of a personal feature (e.g., “My best self would learn not to overthink everything, because right now I brood a lot about everything”).

Finally, all essays were coded independently by two researchers (AC and MMS). Disagreements were resolved by consensus and by consultation with a third researcher expert in the field (RMB). Intercoder reliability was assessed with Kappa coefficients and correlations between coders for all categories. Kappa values ranged from .78 to 1, and correlations ranged from .87 to 1 (see Table 1). These results indicate high levels of agreement [28].

**Table 1:** Kappa values and intercoder correlations.

	Kappa values	Correlation values
**Themes of the texts**		
Personal area		
Positive features	.81	.97
Skills	.78	.87
Health	.88	.90
Academic/professional area		
Intrinsic	.78	.89
Extrinsic	.91	.91
Social area		
Friendship	.78	.91
Family	.91	.90
Partner	.97	.92
Help	.85	.92
Leisure area		
Leisure	.85	.92
**Essay features**		
Positive emotional states	.90	.98
Negative emotional states	.80	.92
Incongruousness	.90	.98
Sensorial details	1	1

*Notes*: For all correlations, *p* < .001. Positive and negative emotional states were subsequently used to calculate the emotional valence of the texts.

### Data analyses

Analyses of the texts were carried out with ATLAS.ti software for Windows (v. 7.5.4). Statistical analyses were conducted using the SPSS software for Windows (v. 24). In order to test the differences between conditions on the content and features of the texts, two multivariate analysis of variance (MANOVAs) were carried out, one for the content themes and another for the text features. Pairwise comparisons using Bonferroni adjustment were conducted when significant differences were found among conditions. To examine the content themes and text features that predicted the change in PA, a stepwise multiple regression analysis was conducted entering the change in PA as dependent variable, and all themes and text features as independent variables. Change in PA was calculated using pre-intervention PA scores and post-intervention PA scores (i.e., change = post-intervention PA—pre-intervention PA), where positive values for change in PA reflected an improvement. Finally, ten parallel multiple mediation analyses (one for each theme) were performed in each condition to test whether the effect of the content of the text on change in PA was mediated by the features of the text, using the procedure described by Hayes [29] from the PROCESS macro (version 2.16), choosing “model 4”. In our proposed mediation models, we included the features of the texts as mediators in the relationship between the themes of the essays and the change in PA. That is, we explored whether the effects produced by the themes of the texts on the change in PA were mediated by how these texts were written. These analyses were carried out for each condition. Bias-corrected bootstrap 95% confidence intervals (CI) based on 5,000 samples were used to assess the specific and total indirect effects. A CI that did not include the zero value indicated a significant indirect effect, implying that the effect of the theme on the change in PA was mediated by the features of the texts. Pairwise comparisons between specific indirect effects were carried out to test whether one indirect effect was statistically different from another through the confidence interval.

For both regression and mediation analyses, the frequency of participants who included each theme and feature in their text was calculated for each condition. This was done as some themes or features were especially uncommon in some conditions. Therefore, if a specific theme or feature appeared in less than 25% of the texts (that is, less than 7 participants of one condition included it in their texts), it was considered that the theme/feature was no representative of the sample on that specific condition, and thus it was not included in the analyses of that condition. For example, sensorial details were not included in the mediation analyses in BPS condition as it appeared in less than 25% of the texts in this condition.

## Results

### Descriptive analyses of the themes

Means and standard deviations of each theme and feature of the texts on the different conditions can be found in Table 2. Generally, the most frequent themes of the texts on the three conditions taken together were *positive features* (M = 2.09, SD = 1.55), *friendship* (M = 1.18, SD = 0.98), and *intrinsic* (M = 0.86, SD = 0.79), and the least frequent ones were *skills* (M = 0.21, SD = 0.57), *health* (M = 0.28, SD = 0.45), *partner* (M = 0.37, SD = 0.58) and *help* (M = 0.32, SD = 0.59). The mean valence of the essays taking all conditions was 2.21 (SD = 2.10).

**Table 2:** Means (M) and standard deviations (SD) for the themes of the essays per condition.

Themes	BPAS M (SD)	BPRES M (SD)	BPS M (SD)	TOTAL M (SD)	ANOVA results	Post-hoc comparisons
**Personal area**						
P. features	1.52 (1.42)	3.04 (1.74)	1.77 (0.99)	2.09 (1.55)	*F*(2, 75) = 8.50, *p* < .001, *η*^*2*^_*p*_ = .185	BPRES > BPAS, *p* = .001; BPRES > BPS, *p* = .006
Skills	0.00 (0.00)	0.40 (0.71)	0.23 (0.65)	0.21 (0.57)	*F*(2, 75) = 3.49, *p* = .036, *η*^*2*^_*p*_ = .085	BPRES > BPAS, *p* = .027
Health	0.19 (0.40)	0.24 (0.44)	0.42 (0.50)	0.28 (0.45)	*F*(2, 75) = 2.04, *p* = .137, *η*^*2*^_*p*_ = .052	*n*.*s*.
**Academic / professional area**						
Intrinsic	0.85 (0.72)	0.80 (0.87)	0.92 (0.80)	0.86 (0.79)	*F*(2, 75) = .16, *p* = .857, *η*^*2*^_*p*_ = .004	*n*.*s*.
Extrinsic	0.52 (0.58)	0.40 (0.65)	0.85 (0.78)	0.59 (0.69)	*F*(2, 75) = 3.02, *p* = .055, *η*^*2*^_*p*_ = .074	*n*.*s*.
**Social area**						
Friendship	1.67 (1.33)	0.88 (0.67)	0.96 (0.53)	1.18 (0.98)	*F*(2, 75) = 5.83, *p* = .004, *η*^*2*^_*p*_ = .135	BPAS > BPRES, *p* = .009; BPAS > BPS, *p* = .020
Family	0.37 (0.56)	0.64 (0.64)	0.88 (0.65)	0.63 (0.65)	*F*(2, 75) = 4.58, *p* = .013, *η*^*2*^_*p*_ = .109	BPS > BPAS, *p* = .010
Partner	0.37 (0.69)	0.16 (0.37)	0.58 (0.58)	0.37 (0.58)	*F*(2, 75) = 3.46, *p* = .036, *η*^*2*^_*p*_ = .085	BPS > BPRES, *p* = .031
Help	0.22 (0.58)	0.20 (0.41)	0.54 (0.71)	0.32 (0.59)	*F*(2, 75) = 2.77, *p* = .069 *η*^*2*^_*p*_ = .069	*n*.*s*.
**Leisure area**						
Leisure	0.56 (1.01)	0.31 (0.74)	0.65 (0.75)	0.49 (0.83)	*F*(2, 75) = 1.74, *p* = .182, *η*^*2*^_*p*_ = .044	*n*.*s*.

*Notes*: P. features = Positive features, *n*.*s*. = not significant.

### Differences between conditions on the content of the texts

Table 2 shows the mean, standard deviations, and the MANOVA results for the effect of condition on the themes of the essays. The MANOVA revealed that, using Pillai’s trace, there was a significant effect of condition on the presence of the different themes, *V* = 0.72, *F*(20, 134) = 3.79, *p* < .001, *η*^*2*^_*p*_ = .36. According to Cohen’s indications [28], the effect size was large (*η*^*2*^_*p*_ > .14). Separate univariate ANOVAs revealed significant effects of condition on *positive features*, *skills*, *friendship*, *family* and *partner*. No significant effects of condition were found on *health*, *help*, *leisure* or on the academic/professional area, neither on *intrinsic* or *extrinsic* themes.

Post-hoc comparisons using Bonferroni adjustment revealed that, regarding personal area, *positive features* were more frequent in BPRES than in BPAS and BPS, and *skills* appeared more frequently in BPRES than in BPAS. Regarding social area, *friendship* was more frequent in BPAS than in BPRES and BPS, *family* was more frequent in BPS than BPAS, and *partner* appeared more frequently in the texts in BPS than in BPRES.

### Differences between conditions on the features of the texts

Table 3 shows the means, standard deviations, and the MANOVA results for the effect of condition on the features of the essays. The MANOVA showed that, using Pillai’s trace, there was a significant effect of condition on the presence of the features of the texts, *V* = 0.22, *F*(8, 146) = 2.31, *p* = .023, *η*^*2*^_*p*_ = .11. According to Cohen’s indications [28], the effect size was moderate (*η*^*2*^_*p*_ > .06).

**Table 3:** Means (M) and standard deviations (SD) for the features of the essays per condition.

Essay features	BPAS M (SD)	BPRES M (SD)	BPS M (SD)	TOTAL M (SD)	ANOVA results	Post-hoc comparisons
Valence	2.96 (2.14)	1.92 (1.98)	1.69 (2.00)	2.21 (2.10)	*F*(2,75) = 2.93, *p* = .060, *η*^*2*^_*p*_ = .072	BPAS > BPS, *p* = .079^1^
Incongruousness	0.19 (0.62)	0.04 (0.20)	0.77 (1.61)	0.33 (1.04)	*F*(2,75) = 3.81, *p* = .027, *η*^*2*^_*p*_ = .092	BPS > BPRES, *p* = .034
Sensorial details	0.63 (1.21)	0.04 (0.20)	0.15 (0.78)	0.28 (0.88)	*F*(2,75) = 3.54, *p* = .034, *η*^*2*^_*p*_ = .086	BPAS > BPRES, *p* = .045
Length	278.78 (92.97)	252.04 (83.05	249.15 (89.85)	260.33 (88.73)	*F*(2,75) = 0.87, *p* = .412, *η*^*2*^_*p*_ = .023	*n*.*s*.

*Notes*: ^1^ = marginally significant, *n*.*s*. = not significant.

Separate univariate ANOVAs and post-hoc analyses using Bonferroni adjustment revealed that the number of sensorial details was higher in BPAS than in BPRES texts, and incongruousness appeared significantly more often in BPS than in BPRES. A tendency to reach significance on the effect of condition on the valence of the essays was found, being more positive in BPAS than in BPS. No significant differences between conditions were found on length.

### Analyses of the predictors of the change in PA: do the themes and features of the texts predict the change in PA?

Three stepwise multiple regression analyses, one for each condition, were used to examine which themes and features predicted change in PA. Variance Inflation Factor ranged from 1.00 to 1.01, indicating no problems with multicollinearity [30,31]. All the themes and features were entered simultaneously. For BPAS, only emotional valence remained as a significant predictor of change in PA (*β* = 0.84, *t* = 2.84, *p* = .009). The model was statistically significant, *F*(1,25) = 8.05, *p* = .009, *R*^2^ = .24, *R*^2^_Adjusted_ = .21, explaining 21% of the variance. By contrast, for BPS, length of the essay (*β* = 0.02, *t* = 2.07, *p* = .050) and *extrinsic* theme (*β* = 3.71, *t* = 3.02, *p* = .006) remained as significant predictors of change in PA. The model was statistically significant, *F*(1,23) = 4.29, *p* = .050, *R* ^2^ = .39, *R*^2^_Adjusted_ = .34, explaining 34% of the variance in PA. In the case of BPRES, none of the variables remained as significant predictors.

### Parallel multiple mediation analyses: do the features of the texts mediate the relationship between the themes of the texts and the change in PA?

Coefficients, Standard Errors (SE) and Confidence Intervals (CI) of the parallel multiple mediations for the significant models can be found in Table 4.

**Table 4:** Coefficients, Standard Errors (SE) and Confidence Intervals (CI) of the parallel multiple mediations for the significant models.

	BPAS condition				BPS condition			
	*Friendship* as predictor		*Partner* as predictor		*P*. *features* as predictor		*Family* as predictor	
	Coefficients (SE)	95% CI	Coefficients (SE)	95% CI	Coefficients (SE)	95% CI	Coefficients (SE)	95% CI
**Indirect effects**								
Total indirect effect	1.25 (0.59)	[0.28, 2.78]	0.86 (0.94)	[-0.96, 2.75]	0.73 (0.97)	[-0.80, 3.29]	3,30 (1,68)	[0.78, 7.89]
S.P. in Valence	0.76 (0.38)	[0.22, 1.94]	0.98 (0.67)	[0.06, 3.29]	-0.14 (0.39)	[-1.39, 0.36]	0.07 (0.38)	[-0.37, 1.39]
S.P. in Length	0.09 (0.39)	[-0.26, 1.40]	-0.01 (0.19)	[-0.61, 0.14]	1.20 (0.80)	[0.13, 3.95]	2.83 (1.62)	[0.50, 7.64]
S.P. in Sensorial details	0.40 (0.38)	[-0.15, 1.23]	-0.10 (0.52)	[-2.13, 0.48]	-	-	-	-
S.P. in Incongruousness	-	-	-	-	-0.33 (0.44)	[-1.82, 0.16]	0.41 (0.62)	[-0.10, 2.62]
**Contrasts**								
Valence–Length	0.67 (0.59)	[-0.18, 1.70]	0.99 (0.72)	[0.06, 3.43]	-1.33 (0.97)	[-4.31, -0.02]	-2.75 (1.73)	[-7.76, -0.29]
Valence–Sensorial details	0.35 (0.59)	[-0.48, 1.80]	1.07 (0.75)	[0.02, 3.40]	-	-		
Length–Sensorial details	-0.31 (0.53)	[-1.28, 0.76]	0.08 (0.56)	[-0.80, 1.64]	-	-		
Valence–Incongruousness	-	-	-	-	0.19 (0.62)	[-0.91, 1.61]	-0.33 (0.71)	[-2.26, 0.57]
Length–Incongruousness	-	-	-	-	1,53 (0.84)	[0.30, 3.96]	2.42 (1.76)	[-0.28, 7.45]

*Notes*: “Incongruousness” was not included in the analyses in BPAS condition as it appeared in less than 25% of the texts in this condition, and the same procedure was followed for “Sensorial details” in BPS condition. P. features = Positive features. S.P. = Specific change

**Fig 1:**
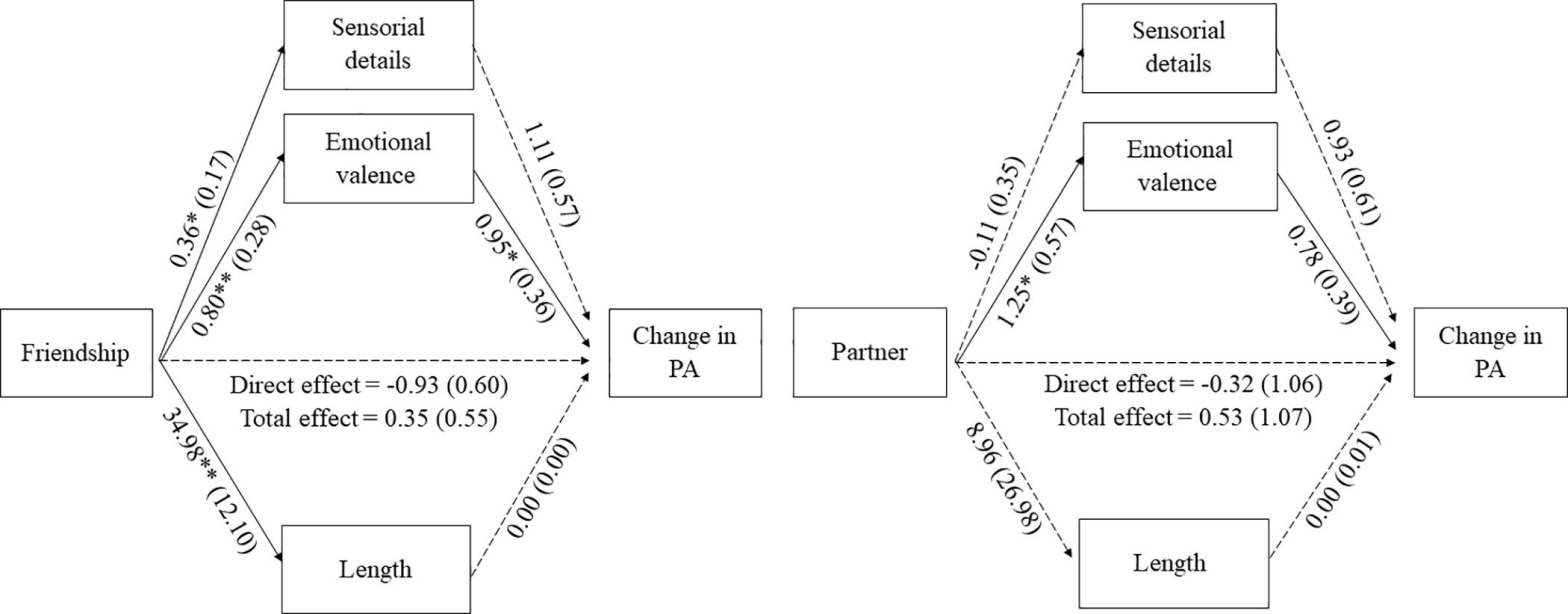
Parallel multiple mediations between content themes and change in PA through features of the texts in BPAS condition. *Notes*: All coefficients represent unstandardized regression coefficients (and standard error in parenthesis). * *p* < .05; ** *p* < .01; *** *p* < .001. PA = Positive affect.

In BPAS condition, there were significant indirect effects of *friendship* and *partner* on change in PA through emotional valence, *b* = 0.76, 95% CI [0.22, 1.95] and *b* = 0.98, 95% CI [0.06, 3.29], respectively (see Fig 1), as bias-corrected bootstrap 95% confidence intervals (CI) for the indirect effects, based on 5.000 bootstrap samples, did not included zero. Neither the total effect, *b* = -0.97, t = -2.02, *p* = .056, nor the direct effect, *b* = -0.55, *t* = -1.02, *p* = 0.319 were significant. No significant indirect effects were found for the rest of the themes and features, as all CI included zero. Thus, results imply that, when participants in BPAS condition wrote about the themes *friendship* and *partner*, they wrote more positive texts (i.e., with higher emotional valence), and that produced higher changes in PA.

Regarding BPRES, no significant indirect effects were found, as all CI included zero and all *p* > .05.

**Fig 2:**
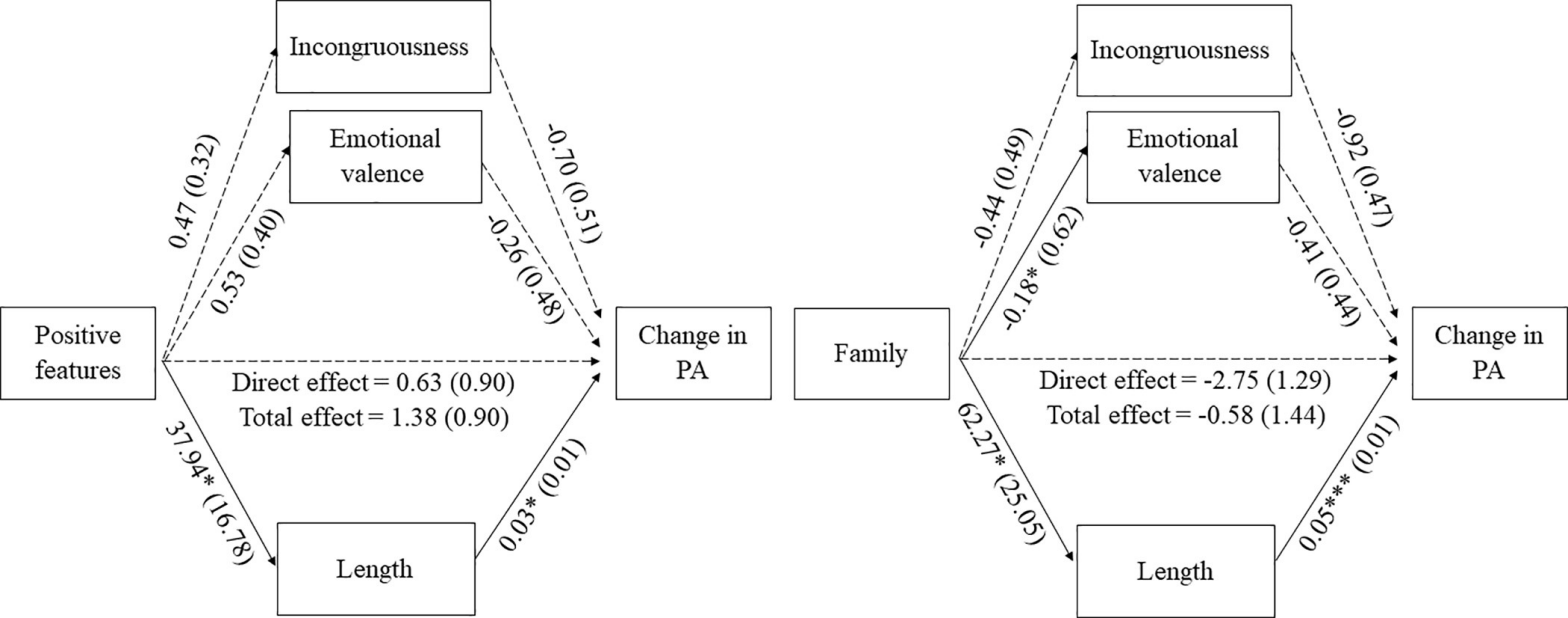
Parallel multiple mediations between content themes and change in PA through features of the texts in BPS condition. *Notes*: All coefficients represent unstandardized regression coefficients (and standard error in parenthesis). * *p* < .05; ** *p* < .01; *** *p* < .001. PA = Positive affect.

For BPS, there were significant indirect effects of *positive features* and *family* on change in PA through length (i.e., number of words), *b* = 1.20, 95% CI [0.13, 3.95], and *b* = 2.83, 95% CI [0.50, 7.64] respectively, given that bias-corrected bootstrap 95% confidence intervals (CI) for the indirect effects, based on 5.000 bootstrap samples, did not include zero (see Fig 2). Again, neither the total effect, *b* = 1.38, t = 1.52, *p* = 0.141, nor the direct effect, *b* = 0.63, *t* = 0.71, *p* = 0.488 were significant. No significant indirect effects were found for the rest of the themes and features, as all CI included zero. These results suggest that, when participants in BPS condition wrote about their *positive features* or *family*, they wrote longer texts, and that produced higher changes in PA.

## Discussion

This study showed that, despite the similar effects produced by writing about one’s best self in the past, present or future on positive mood [13], these interventions respond to different underlying mechanisms. The procedure of the included PPIs was identical (to write about the best version of oneself), and the only difference between them was the time frame in which participants had to focus on: their past, present or future. Notably, significant differences were found in the content on the compositions depending on the condition. When writing about their best past self (BPAS), participants more frequently included their social relationships with their friends than the other conditions. In addition, they added more sensorial details than the ones who wrote about their present self, which goes in line with previous studies that suggest that recalling past events exhibit more sensorial details than imagining future events, as the latter needs more mental work to supply these [32,33]. In the case of participants who wrote about their best present self (BPRES), they talked more frequently about their personal area, including their *skills* more often than in the past condition, and their *positive features* more often than the rest of the conditions. Lastly, when participants wrote about their best possible self in the future (BPS), their texts focused more on their familial relationships, being their *family* more frequently included than in the past condition, and their *partner* more frequently than in the present condition. In addition, they included more incongruousness in their essays comparing with the present condition. Some of these results are in consonance with previous studies about self-descriptions, which showed that participants’ descriptions of their current self are more focused on oneself, followed by their past self-descriptions and least of all their future self-descriptions, which were more socially oriented [34].

As regards to predictions of change in PA, emotional valence arose as a significant predictor of change in PA in the BPAS texts, whereas the length of the essay and academic or professional theme extrinsically motivated remained as significant predictors of change in PA for participants in the BPS condition. That is, when writing about their best past self, the more positive the compositions participants wrote, the better results on their levels of positive mood they obtained. Conversely, when writing about their best possible self in the future, the more words participants wrote, or the more they included the extrinsic academic or professional theme, the more benefits they achieved on their positive mood levels. In the case of present condition, none of the variables remained as significant predictors.

With respect to mediation analyses, significant indirect effects of *friendship* and *partner* on PA change through emotional valence in the case of BPAS were found. For the BPS condition, significant indirect effects of text length on PA change through *positive features* and *family*. In other words, when participants wrote about their past self and talked about their relationship with their friends or their partners, this led to greater positivity in their texts, which produced improvements in their levels of positive mood. In the case of participants who wrote about their best possible self in the future, when they focused on their own positive features or their relationships with their family, this produced longer texts, which led to better results in their levels of positive emotions. In the case of participants who wrote about their best current self, no indirect effects were found.

Based on these results, it is possible to conclude that there are differences in the content and form of the compositions of the three PPIs as well as their underlying mechanisms: even that all of them consisted of writing about their best selves, the themes and features of their essays were different, and the factors that predicted and mediated the change in the level of positive mood were also different. It seems that positive emotional valence in combination with social themes as *friendship* or *partner* play an important role in the BPAS condition, whereas the length of the essay combined with *positive features* or *family* have an impact on the efficacy of the BPS condition. It is worth to note, however, that the analyses did not find significant results on the BPRES condition.

These results can have important implications. Approaching to disentangle the working mechanisms of psychological interventions can help practitioners to use the interventions to their most potential. In this sense, the results obtained in this specific study may help to boost the effects of the interventions by, for example, modifying or highlighting specific features in the instructions. Since emotional valence seems to be a key component of the BPAS condition, it could be beneficial to encourage participants to include as many positive emotional states as possible when they write about their best past self. However, some participants can feel frustrated if they are not able to naturally include positive emotional states when they are asked to do so. In this case, and based on the results on the mediation analyses, emphasizing the social area (writing about their friends or partner) could indirectly boost the efficacy of this PPI. Following the same rationale, the length of the text is an important factor in the future condition. It is possible to encourage participants to write down as much as possible. However, it is not feasible to know *how much* they should write, and it is possible that some discomfort reactions could arise in a participant who does not accomplish to write as much as asked. In the same manner, after mediation analyses results, asking participants to focus on their positive features and family relationships in their texts could indirectly amplify the efficacy of the intervention.

This study has some limitations that are necessary to address. First, the sample included was considerably young (M = 20.23, SD = 4.10). Even though this is not a limitation *per se*, including a sample with more heterogeneous age could have helped us to produce more generalizable results. In addition, it would be highly interesting to conduct future studies in a more heterogeneously aged sample, in order to explore whether older participants show the same pattern as the younger ones. Second, we were not able to find which mechanisms underlie in the efficacy of writing about one’s present best self (BRES). It is possible that the term “present self” seemed too broad to participants, which led to an excessively heterogeneous time range to find significant results. Previous research has found that there are significant differences between recalling near and far past events, as well as between imagining near or far future events [33,35]. Participants writing about their best self in the present could have focused on their present moment, but it is also possible that some of them included near past or even future time frames, as it was not predefined in the instructions, thus different processes may have been affecting on this condition. Future studies should explore this condition in more detail, either encouraging participants to focus on a specific time frame or exploring which time range they included in their texts.

This work has been the first attempt to study which are the underlying mechanisms of the BPS intervention and the two variants derived from it, and the role that these mechanisms have on their efficacy. There is evidence about PPIs being efficacious resources to improve wellbeing over different populations, but the research about the mechanisms that produce those benefits is still in its infancy [5,12,13]. Hence, there is still much more to investigate in this regard. This study helped to shed light on the importance of the idiosyncratic features of PPIs in order to better understand how they work. Results obtained provide a richer knowledge about the process that takes part when participants practice the BPS intervention and its temporal variants. This deeper understanding can be a powerful tool to increment their efficacy by, for example, modifying the instructions of the exercises. We encourage researchers to continue the investigations on this topic, as a better knowledge about why and how PPIs work will help psychologists and other professionals to make the most of these valuable resources.
